# Biological species is the only possible form of existence for higher organisms: the evolutionary meaning of sexual reproduction

**DOI:** 10.1186/1745-6150-5-14

**Published:** 2010-03-22

**Authors:** Victor P Shcherbakov

**Affiliations:** 1 Institute of Problems of Chemical Physics RAS, Chernogolovka, Moscow Region, 142432 Russia

## Abstract

Consistent holistic view of sexual species as the highest form of biological existence is presented. The Weismann's idea that sex and recombination provide the variation for the natural selection to act upon is dominated in most discussions of the biological meaning of the sexual reproduction. Here, the idea is substantiated that the main advantage of sex is the opposite: the ability to counteract not only extinction but further evolution as well. Living systems live long owing to their ability to reproduce themselves with a high fidelity. Simple organisms (like bacteria) reach the continued existence due to the high fidelity of individual genome replication. In organisms with a large genome and complex development, the achievable fidelity of DNA replication is not enough for the precise reproduction of the genome. *Such species must be capable of surviving and must remain unchanged in spite of the continuous changes of their genes*. This problem has no solution in the frame of asexual ("homeogenomic") lineages. They would rapidly degrade and become extinct or blurred out in the course of the reckless evolution. The core outcome of the transition to sexual reproduction was the creation of multiorganismic entity - biological species. Individual organisms forfeited their ability to reproduce autonomously. It implies that individual organisms forfeited their ability to substantive evolution. They evolve as a part of the biological species. In case of obligatory sexuality, there is no such a thing as synchronic multi-level selection. Natural selection cannot select anything that is not a *unit of reproduction*. Hierarchy in biology implies the functional predestination of the parts for the sake of the whole. A crucial feature of the sexual reproduction is the formation of genomes of individual organisms by random picking them over from the continuously shuffled gene pool instead of the direct replication of the ancestor's genome. A clear anti-evolutionary consequence of the sexuality is evident from the fact that the genotypes of the individuals with an enhanced competitiveness are not transmitted to the next generation. Instead, after mating with "ordinary" individuals, these genotypes scatter and rearrange in new gene combinations, thus preventing the winner from exploiting the success.

**Reviewers:**

This article was reviewed by Pierre Antoine Pontarotti, Michael T. Ghiselin (nominated by Dr. Juergen Brosius) and Emanuel Tannenboum (nominated by Dr. Doron Lancet)

## Preamble: declaration of the holistic viewpoint

The Universe consists of discrete entities: elementary particles, atoms, molecules, planets, stars, galaxies. That is there are a limited number of configurations of matter that are fairly stable and lasting, the intermediate ones being volatile. The Universe is structuralized. It means that it is far from thermodynamic equilibrium; it contains information; it exists. The existence of the Universe depends on the mutual affinity of its constituents, their ability to interact with each other, thus resisting the general aspiration for evenness. Initially, the Darwinian natural selection, acting by the accumulation of tiny heritable changes, was supposed to produce an even continuum of the living beings. This expectation was never corroborated. The biological world follows the same global principle: organisms, populations, species, ecosystems are discrete, relatively stable entities, the intermediate configurations being volatile. Biological evolution cannot retain everything that randomly emerges. The stability of the biotic entities is determined not merely by their physical durability but by their expedient behavior especially. They are organizations with the function of survival. The Universe evolves via the interaction and cooperation of the entities, whence its complexity and hierarchical structure come from. The major transitions in biological evolution (macromolecular replicator → prokaryotic cell → eukaryotic cell → multicellular organism → biological species) are also the steps of cooperation [1]. Though a complex entity consists of the other simpler ones, it is not just an aggregate of the included entities. It is a qualitatively new form of existence; it is an organization of a higher rank. Hierarchy in biology doesn't mean just complexity or heterogeneity. It implies a functional predestination of their parts for the sake of the whole. Survival of the parts crucially depends on survival of the whole. Hence, constituent entities are to be included into the higher entities only in an appropriately transformed configuration. The operating principles of the organization of the higher rank are not necessarily related to or derivable from the properties of the parts or to their internal operating principles. That is the principles organizing an upper rank are novelties. They are not necessarily predictable from the rank below. On the other hand, the organizing restrictions of the living entities, being emerged as *a frozen chance*, cannot be deduced from any general principle or law. They can be understood only retrospectively, in the context of their history. The above statements imply that the evolution of a higher entity cannot be adequately presented as self-sufficing evolution of its constituents. The prosperity of the whole is the vector of selection for the constituent entities.

## Introduction

A great number of theoretical models have been suggested to explain the widespread occurrence of sexual reproduction (reviewed in [2-5]). Nevertheless, the papers on this topic continue to appear, beginning frequently with a phrase: "Sexual reproduction is a paradox, an enigma, a mystery", and the like. If a common and ubiquitous phenomenon looks like a paradox, we most likely look at it from the wrong position and do not comprehend something fundamental. I guess that this wrong position is the perception of evolutionary process as a value in itself. When searching for possible evolutionary advantages of sexual reproduction, we (explicitly or implicitly) regard it as not a final result of evolution but its instrument. The Weisman's idea that sex and recombination provide variation for the natural selection to act upon dominates in most of the discussions. But is the ability to evolve rapidly is unambiguous advantage for an entity? Here I am going to substantiate the heretical idea that the main advantage of sexual reproduction is the opposite: the ability to counteract evolution. Natural selection selects those who survive. However, to evolve and to survive are not the same things. Rather, they are opposite things. To evolve means to change, while to survive means to persist. And persistence means also resistance to further changing. We used to think that organisms die and species become extinct because of poor adaptation to the environment or because of losing in competition with other organisms and species for substances and energy. We give little significance to the fact that organisms inevitably die even in the most favorable environment, in the absence of any competition, with an abundance of energy and substance. They die because of imperfection of their homeostatic mechanisms, because of their limited ability to resist the universal disruptive force - increase of entropy. I know exactly that I will die. Most probably, I will not be killed by my rival or die of starvation. Moreover, the physicians and surgeons will try to prolong my life by all available means. Yet they will fail, and entropy will be the victor. Notably, the same entropy is a driving force of evolution [6]. Not only those who die do not survive but also those who evolve. The entities that change rapidly disappear rapidly and for this reason they are not observed among fossils and now-living organisms. (Still many are preoccupied with the possible shortage of the mutation load, *e.g. *[7]).

### Survival by means of reproduction

Organisms are complex and highly organized systems that are far from maximum entropy. "In defining 'organization', I will use the conception of Denbigh [8]: an organized system is a complex system that can perform certain functions by virtue of its particular assemblage of parts. Organized systems must be distinguished from the ordered ones. Neither system is random; but the ordered systems are generated according to a simple algorithm and therefore lack complexity, whereas the organized systems must be assembled element-by-element according to an external program or plan. Their structures are aperiodic without being random. Hence, organization is complexity endowed by function. It is not random due to design (machines) or selection (organisms), rather than to the *a priori *necessity of crystallographic order [9].

The homeostatic mechanisms, aimed at keeping the system in the stationary state, are not perfect; therefore, no individual can avoid a thermodynamic equilibrium, which is death. Living systems bypass the thermodynamic limit by reproduction. The essence of reproduction phenomenon is not increasing the number of organisms, not just multiplication, but renewal, replacement the old, worn-out bodies by the new ones. Being unable to retain their physical structures perpetually, organisms retain the information needed for their continuous regeneration. Maintenance of information rather than the bodies is the most distinctive attribute of life demarcating it from abiotic existence. The reproduction of the live systems on the basis of genetic information is the contents of life. Ideally, the copying genetic information should be precise. Otherwise, the goal of the maintenance is not accomplished. In reality, however, the copying cannot be errorless. Extra copying (multiplication), coupled with selection, guarantees a precise reproduction, but on the other hand, the possibility of evolution thus emerges.

In the case of a unicellular asexual organism, the reproduction includes the doubling of DNA and the regeneration of all other components of the cell. The daughter cells inherit the entire genome and the entire equipment for the reproduction of the mature cell. It is important to realize that without this equipment the genome would be just a dead text with nobody to read it. I would like to stress that neither separate genes nor the whole genome represent the living entity. The biological form of existence begins with a cell. Only a cell, not any of its constituents, is "organization with the function of survival" [10]. The overwhelming majority of the multicellular organisms have a unicellular stage (generative cell) in their life cycle. In the case of asexual multicellularity, there is little change in the method of information transmission between the generations except the information itself contains a more sophisticated ontogenetic plan.

With sexual reproduction, the situation is changed drastically. An individual organism cannot reproduce itself acting alone. Genome of the generative cell (zygote) is formed via a fusion of two different cells, maternal and paternal. These links generate a new entity - the biological species. A generation of the sexual population becomes a self-reproducing unit. The daughter generation inherits the gene pool distributed among the multiplicity of the unique zygotes with their unique developmental programs.

Natural selection selects all that survives, *i.e. *mainly and primarily the old-established things [11,12]. It is not an opinionated selectionist, it is a thoughtful breeder. Selection is just checking the perfection of the homeostatic mechanisms. The function of natural selection is largely conservative. Without this conservatism, only chaos would be possible. Novelties have a chance to be selected only if they improve or at least do not worsen essentially the homeostasis. Adaptation to the environment is an important element of the homeostasis, but the major vector of selection is the internal perfection and harmonization of the system that would guarantee its precise and reliable reproduction. The precise and reliable reproduction, when achieved, entails stasis,*i.e. *a halt of evolution and salvation of the lineage.

I am going to develop here the following thesis: The stable existence of the higher organisms is only possible in a form of the higher order individual, biological species. I accept here the Mayr's definition of biological species: "Species are groups of interbreeding natural populations that are reproductively isolated from other such groups" [13]. All asexual organisms are the simplest ones, while obligatory sex is a property of the higher animals. Simple organisms (like bacteria) reach the continued existence on account of high fidelity of individual genome replication. In organisms with a large genome and complex development, the achievable fidelity of DNA replication is not enough for the precise reproduction of the genome. Such species must be capable of surviving and must remain unchanged in spite of continuous changes of their genes. This goal has no solution in the frame of asexual ("homeogenomic") lineages. They would rapidly degrade and extinct or blurred out in the course of the reckless evolution. The core outcome of the transition to sexual reproduction was a creation of multiorganismic entity - biological species. This transition, like all other major transitions in evolution, is cooperation. Individual organisms forfeited their ability to autonomous reproduction. It implies that the individual organisms forfeited their ability to substantive evolution. They evolve as part of the biological species. It is worthy to note that the renunciation of one's freedom and independence for the reliability and wellbeing is a common step in progressive evolution. Essentially, the robust genomes created via sexual reproduction are not necessary identical to each other, so the organisms of the same biological species are not identical genetically ("allogenomic" lineages). Being unable to retain precisely their genotypes, higher organisms retain the information needed for continuous regeneration of their phenotypes.

### Apparent oddity of sex

Sexual reproduction is so ubiquitous that even many biologists rarely make up their mind to ask what is the evolutionary process that gives rise to this complex, troublesome, and risky way of multiplication. Asexual reproduction is obviously simpler and more effective in transmitting genes from ancestor to progeny. With asexual reproduction (contrary to sexual one):

1) Each individual produces progeny;

2) There are no problems related to a search for a mating partner;

3) Each individual transmits all of its genes to the offspring (not half);

4) Once created, good combinations of genes (optimal genomes) do not disperse in the next generation, but are transmitted to all of the offspring of a given individual.

The high cost of sexual reproduction [14-16] was clearly realized first by A. Weismann [17]. The obvious advantages of asexuality are in a drastic contradiction to the intuitively evident biological importance of the sex. Some or other forms of the genetic information shuffling are known in organisms of all levels of organization, from viruses to humans; almost all eukaryotic organisms are sexual [18-20], while mammals, in which both male and female gametes are necessary for the successful initiation of development [21], lost the ability to bring on asexual reproduction irreversibly. Biologists know very well what a dominant role reproductive behavior plays in the life of animals. It may be that Freud is not absolutely right when he reduces the entire human psychology to the expression of the sexual instinct, but he is right to a great extent. All this fuss serves to provide a meeting of the male and female chromosomes, to shuffle their information and create gametes with unique gene combinations. The importance of this information exchange must outweigh all the shortcomings and complications of sexual reproduction. Paradoxically, potentially lethal DNA damage (double-strand break) is used to initiate recombination during meiosis [22].

For a long time, the problem of emergence and maintenance of sexual reproduction attracted little attention from evolutionists. The matter probably seemed too obvious. No one doubted Weismann's idea that sexual reproduction, creating genetic variability, produces material for natural selection and enhances the evolutionary potential of the species. A possibility of the acceleration of evolution at amphimixis was quantitatively substantiated by Fisher [23] and H.J. Muller [24]. The conception of the evolvability is still popular among population geneticists. It is frequently assumed that the capability for rapid and diverse evolution is a positive trait supported by natural selection, while a shortage of the evolutionary potential is fraught with extinction. The notion of evolvability as a selectable trait is in evident contradiction to the known efforts of evolution aimed at creating genetic stability of organisms and lineages [25]. It is obvious that the evolvability cannot be easily taken as a species homeostatic mechanism. Direct selection for evolvability is impossible conceptually [25,26], so the transition to sexuality needs another explanation, independent of the evolvability. Though sexual reproduction and genetic recombinations are a source of combinative variation in populations, they do not produce new alleles but only new combinations of the extant ones, which are, moreover, doomed to be destroyed in the next generation. If to think that the sexual reproduction was invented for acceleration of evolution (Lamarckian thought, by the way) than the continuous shuffling of the genomes (heedless of their merits) looks more than strange. I think we should not assume special mechanisms for the acceleration of evolution created by evolution. These would be suicidal mechanisms. A species with accelerated evolution would not exist long. All the organisms populating our earth today belong to species resistant enough to further evolution. Evolution is inevitable because the systems created by evolution for protection against evolution, species homeostasis, are not absolutely perfect, and the entropy pressure overcomes them now and then. All the species are capable of evolving just because they originated from the ancestors that were capable of evolving and inherited their imperfection, their "original sin". It is hard to avoid evolution.

Of course, the alternative to evolve or become extinct, both of which lead to the disappearance of the species, is not indifferent for the biosphere. If the first living cell happened to be absolutely perfect and was able to have infinite life but unable to change, there would be nothing more than those cells. (The like took place during the first two billions years of the life history when the Earth was populated solely by microbes [27]. But this trivial thought does not prove and does not mean that evolution can create special mechanisms for the acceleration of evolution, though there is little doubt that the enhanced evolutionary potential could emerge as a by-product of the development of other systems and mechanisms (including sexual reproduction [28]). A complexity of the biological systems, which increases during evolution, obviously increases a range of possible evolutionary novelties. But the same complexity broadens an opportunity for creation of the more perfect organization that may occur more resistant to the enhanced entropic challenges. Such is the dialectics of the evolution phenomenon.

Genetic polymorphism increases the morphogenetic homeostasis of a species [29]. Genetic diversity of populations may be valuable in itself, for example, by providing an ecological plasticity and efficiency of intraspecies interactions and cooperation [30]. It may play a crucial role in creating the stable dynamics of a population under varying environmental conditions [31,32]. An adequate explanation of the meaning of sexual reproduction should be searched in the context of the evolutionary stability.

During the last three decades, the problem of the evolution of sexual reproduction got topicality. In numerous, mainly theoretical, works, the various hypotheses on the origin and maintenance of sex and genetic recombination were presented [28,30,32-52]. Some of these works agree with the evolution-accelerating role of sexual reproduction, whereas others reveal its conservative, stabilizing function. Sexual reproduction is such a powerful acquisition of evolution (it is sufficient to say that it was appearance of a new unit of selection, an individual of a higher rank [53-64], that its effects and consequences could be observed in the study of very different aspects of life. In numerous theoretical analyses and computer simulations, both the selective advantages and shortcomings of sexual reproduction, as compared to the asexual one, were observed. The results of a few experimental works were diverse [46-48]. In recent large-scale experimental evolution studies on *Caenorhabditis elegans *[52] it was shown that outcrossing is favored in populations subject to experimental evolution both under conditions of increased mutation rate and during adaptation to a novel environment.

I think the issue is not that the models demonstrating high adaptive value of sex are wrong or right. I deeply doubt that the direct comparison of sexual and asexual populations in the evolutionary aspect is an eligible approach. Sexual and asexual individuals have different biological status. An asexual individual is a self-sufficing sovereign player on the stage of life, whereas a sexual, especially obligatory sexual individual is a law-obedient citizen of the multi-organismic realm - biological species. There are two most essential, though interdependent, differences: first, a sexual individual, in contrary to an asexual one, is not a self-reproductive entity; second, its behavior must be aimed at the survival of the species, not exclusively at its own survival and multiplication. As a consequence, the sexual and asexual populations must use different evolutionary strategies. In particular, the competition between the individuals must have different biological consequences: replacement of one lineage by the other (in case of asexuality) and working out of the species organization, development of species robustness (in case of sexuality). Both can successfully resist extinction and reach stasis but by different routes: via high fidelity of genome maintenance (in case of asexuality) and by the creation of diversity and genotypic plasticity (in case of sexuality). Adaptability and variability are instruments of evolution, whereas its ontological content is the creation of the stable forms that are resistant not only to extinction but to further evolution as well. When the population geneticists estimate the fitness and the surviving times of the populations using mathematical models and computer simulations, they are never interested in how much a survived population retained its self-identity and was not sacrificed to conformism.

### Sexual reproduction

Notionally, one may think that in order to persist in time, a biological system must have homeogenomic reproduction, *i.e*. the genomes of the ancestors and descendants have to be identical. In case of single-cell organisms, this stipulation is observed. For example, the rate of spontaneous mutations in growing cells of *Escherichia coli *is about 0.003 alterations per genome per replication [65,66]. Other microorganisms, including eukaryotic ones, have a similar or a higher fidelity of genome replication. Asexual lineages, being either unicellular organisms or organisms with a small number of germ-line cell divisions, survive due to the very high fidelity of DNA replication, which is enough for the reliable self-reproduction. But the replication with high fidelity is a costly process. It looks probable that the attained fidelity of DNA replication per base pair per cell division is close to the maximum [67,68] (but see [69]. Consequently, the per-generation rate of mutation in organisms with a large genome and a large number of germ-line cell divisions is very high, up to three orders of magnitude higher than, for example, in yeast [66-69]. *I.e.*, genomes of higher organisms are not reproduced with high fidelity. For example, in man the number of mutations per zygote is 60 or even more [66,70-73]. The mutational deluge menaces to destroy both homeostatic mechanisms of the organisms and the evolutionary stability of the species. It is hard to imagine how such a dissipating action of mutagenesis can be compatible with life. We have to admit, however, that such species are capable of surviving and remain unchanged in spite of continuous changes of their genes. Looks like a logical error! But, as Andre Lwoff uttered: "Problems do not exist in nature. Nature only knows solutions." The solution was: "Sexual reproduction" or better to say: "Biological species".

### Cohesion

The transition to sexuality is not merely a change of a mode of reproduction but ascension to a new quality. Sexual populations are coherent systems. Cohesion is a very complex phenomenon and an important notion, the sense of which can be elucidated by comparison of sexual and asexual reproduction. With asexual reproduction, a parent transmits all its genes to an offspring. Hence, the offsprings are genetically identical to the parent. The individuals of the same population do not exchange their genes and are reproductively isolated. All the progeny of one individual form a clone of genetically identical individuals. The lineage of the clone presents a tree with a single progenitor; the lineage of each individual is a monomeric line of the ancestors. If a mutation appears in the individual, it will be transmitted to all its offsprings, but it cannot be transmitted to other clones, *i. e. *genes are transmitted only vertically. If a population consists of different clones, it is genetically heterogeneous. A competition between the individuals is simple: it is reduced to the competition between the clones. The clones with selective advantages replace all the rest. Strictly saying, the reproductive isolation of asexual lineages is not complete, and horizontal gene transfer plays essential role in evolution of prokaryotes and single-cell eukaryotes [74-76]. The capacity to exchange genetic information enables to asexual populations to support genetic diversity resulting, in some cases, in important outcomes, such as antibiotic resistance.

Now consider a population with sexual reproduction. Though the genetic material exchange and recombinations occur in haploid organisms, including bacteria and other prokaryotes, the genuine sexual reproduction is a prerogative of the eukaryotes and it is coupled with the alternation of the diploid and haploid phases of a life cycle. In the most complete form, it is presented in vertebrates, in which the haplophase is reduced to gametes, oocytes (eggs) and spermatozoids.

Life of the individual begins from the fusion of the male and female gametes and formation of a zygote, in which two sets of genes of the different origin are combined. During meiosis, paternal and maternal chromosomes are randomly distributed between the two haploid cells. In addition, they exchange their genetic information, also randomly. As a result, each generative cell obtains a unique set of the genetic information of the dual origin, half paternal and half maternal. Consequently, each parent transmits to the offspring only one half of its genetic information picked up randomly. A lineage of the individual is a dichotomous tree receding into the past. The lineages of the different individuals are interwoven into a multimeric network. A shared population gene pool is formed. An individual organism has no ability to reproduce its genocopy. *A superindividual unity - biological species emerges*. The species category acquires a distinctive biological status instead of a more or less arbitrary assembly of the similar individuals [77]. Strictly saying, there is no reproduction of an individual organism. Its existence is ephemeral. It emerges on one occasion in the history of the biosphere and never appears again. Species becomes genuine reproductive units.

Given the biological species is a superindividual; an interesting consideration may be useful. The number of organisms comprised by a species can be assimilated to the "megaploidy". This "megagenome" with its billion of chromosomes enables the species to create huge genetic diversity, which is not accessible for the asexual lineages. The emergent species property that follows is *genotypic plasticity *- ability to change reversibly the genotypic configuration of population gene pool in different environments. A crucial feature of sexual reproduction is formation of genomes of individual organisms by random picking them over from the continuously shuffled gene pool instead of the direct replication of the ancestor's genome. Here is the solution of the enigma, how species are capable of surviving and remain unchanged in spite of continuous changes of their genes. In essence, this "picking over mechanism" is the intrinsic selection for perfection of the organization, which begins long before the organism will be tested by the environment.

### Intrinsic selection

The existence of a haploid phase in a life cycle of a species is extremely important for maintenance of the species robustness. In higher animals, this phase is reduced to the generative cells. During the haploid phase, new gene combinations are checked for vitality of the basic cell functions. These new gene combinations are created by meiotic recombination preceding the formation of the gametes. The recombination includes random exchanges of the DNA sequences between the homologous chromosomes and random segregation of the homologs into haploid daughter cells. Even at the perfectly random distribution of the exchanges along chromosomes, the recombination may lead to some interesting and very important statistical consequences. If each homologous chromosome bears one new mutation, the crossing-over may create two daughter chromosomes, one with the two mutations, and the other with no mutation at all. Instead of two chromosomes with one error each, we have one chromosome without errors and one chromosome with the two errors. This is true in a general case also. For any number of new and inherited mutations, their recombination results in an increase of the probability both for zero class (without mutations) and the class with a mutation number more than the average. Random segregation of homologous chromosomes leads to the same consequences at the level of the genome as a whole. All of these events occur just before the formation of haploid gametes, where the mutations are not protected by the normal alleles. The gametes with the multiple defects will most probably be of low vitality and die, sacrificed for the good (unchanged) ones. In addition, the cells with bad mitochondrion undergo apoptosis during oogenesis [78]. The purifying action of the recombination may not be fully random. Biased gene conversion directed against the most common type of genetic damage can substantially reduce the mutational load [79]. Here we have a phenomenon of a cosmic scale: one random stochastic process (recombination) makes not quite random the results of the other stochastic phenomenon - distribution of the mutations among chromosomes. What does it look like? A discard? Or Maxwell's Demon who works, however, blindly like Boltzmann's Demon. Usually, accentuation is made on the fact that sexual reproduction enables a population to get rid of the harmful mutations [33,35,80,81]. In the present context, another side of the coin, an opportunity to restore the initial genetic text is more important.

To what extent is the genetic recombination successful in carrying out this Maxwell's Demon function? The rate of recombination is different in different species. It is interesting, however, that the less is the site separation and the more the recombining chromosomes are dissimilar the higher is the specific rate of recombination. Because of this high negative interference [82,83], the probability for the restoration of the initial (wild-type) allele remains high even with multiple differences between the recombining chromosomes. The famous phenomenon of the multiplicity reactivation in bacteriophage T4 [84] is a remarkable model example of such a recombination effect, when the viable phage particles are formed after infection of the bacterial cell with two or more phage particles each of which bearing several lethal damages. The effect may not be limited by restoration of the wild-type sequences. Functionally robust genomes may be produced via combination of different mutated sequences. An important observation was made by Drummand *et al*. [85]. They directly demonstrated that recombination of related proteins preserves function with a higher probability than random mutation. It means that intragenic recombination is capable not only of restoring the original DNA sequence but also of restoring gene function on the basis of a new DNA sequence.

The intrinsic selection is the major one. It acts as a sole mode during fertilization and embryogenesis and it is probably most important during maturation as well. This selection is difficult to interpret as a competition between the perfect and less perfect entities. One would think that they survive or die according to their own merits, irrespective of the presence of the other entities. The intrinsic selection is quantitatively most effectual and economical. The processes operating during spermatogenesis and oogenesis may also present a kind of intrinsic selection. The overwhelming majority of the generative cells and their predecessors undergo programmed cell death (see *e.g. *[86-88]). At least partly, this mass suicide is aimed at selecting genetically robust germ cells. For example, in the testis of wild-type mice, the mutation rate was shown to decline during spermatogenesis: on the way from type A to type B spermatogonia, the mutation frequency is reduced by a factor of five. This reduction coincides with a wave of apoptosis of the type A cells [89]. *I.e., the heavily mutated cells mainly commit suicide*.

### Muller's ratchet

A gene consists of thousands or even millions of nucleotide pairs. On a structural level, any change in the nucleotide sequence such as base substitution, insertion or deletion of one or several base pairs, and inversion are direct mutations of this gene. On the functional level, not all structural mutations lead to a change of the functional activity of the gene and, correspondingly, to a change of the phenotype, though a considerable part of them do change (most frequently deteriorate) gene function. On the structural level, mutations are reversible. Though the rate of direct and reverse mutation, generally speaking, may not be the same in each particular case, on the average it can be assumed that direct and reversed mutations occur with equal probability, *i.e. *on the structural level mutagenesis is a reversible microprocess. On the functional level, the situation looks quite different: a reversion of function is thousands times less probable than its damage. This means that the mutagenesis on the functional level is virtually irreversible. Each mutant gene has a higher probability (by several orders of magnitude) to get one more direct mutation than to reverse. Hence, on the functional level, mutagenesis is an entropic macroprocess with the same formal description as molecular diffusion.

Since mutations may be harmful, the consequences of this irreversibility must be fatal. The situation is saved by the very high fidelity of gene reproduction, so that the mutations occur rarely. Nevertheless, a mutated gene will either be eliminated, being too harmful, or inherited, but never returned to the initial state. Such is a situation in an asexual population. This shortcoming of the asexual reproduction was first realized by G. Muller and was finally formulated as "ratchet" [90]: "an asexual population incorporates a kind of ratchet mechanism, such as it can never contain, in any of its lines, a load of mutation smaller than that already existing in its at present least-loaded lines." If, as a result of a genetic drift, the least-loaded line is lost, the ratchet will make a click. The ratchet never rotates back. Attention to this discovery was later drawn by Felsenstein [33], who confirmed in a computer simulation study the reality of Muller's ratchet mechanism. In recent work by Neiman *et al*. [91], direct evidence for a link between asexuality and mutation accumulation was obtained, which implies that mutational buildup could be rapid enough to contribute to the short-term evolutionary mechanisms that favor sexual reproduction.

It is easy to imagine how an asexual population, being pushed by entropy (mutagenesis) and attracted by adaptive advantages, moves through a labyrinth of the future and finds itself, after all, in a blind alley without any opportunity to return, if only to the previous furcation. After each step, evolution of the asexual population burns its bridges behind it. Sexual reproduction is not so careless. Since even mutations in the same gene, being functionally similar, are usually located in different sites, a principal opportunity to restore the initial genotype via recombination always remains. *Owing to the recombination, the intraspecies variations are potentially reversible*, as if sexual populations have the Ariadne's clew, so that the blind alleys are not fatal for them.

### Tempo of evolution

The clones of an asexual population can evolve independent of each other, while the sexual population has to evolve as a whole. Intuitively, it looks obvious that the evolution of a population or a species as a whole cannot be rapid, even from "the gene-centered view of evolution". To become an irreversible event, a new allele, even if it is beneficial, must be fixed. The beneficial mutations are extremely rare events. Hence, the owner of a rare new allele will mate with a partner without this allele, so that in the progeny, this useful allele will be in heterozygous state, *i.e. *most probably it will not be expressed phenotypically, thus avoiding positive selection. At sexual reproduction, a multiplication of the genotypes is a square function of their density within a population; therefore, rare types cannot be multiplied even if they have very high fitness (the cost of rarity) [62].

Sexual reproduction and recombination create genotypic diversity of organisms. I think, however, that it would not be wise to see a positive meaning of the diversity in accelerating evolution. In the form of genotypic plasticity, it emerges during "species ontogenesis" as an ability to resist evolution [29,30]. I like the reasoning by Robson *et al*. [32] about a distinction between what they described as "aggregate uncertainty" (in which the reproductive output in each generation is correlated among the individuals in a population) and "idiosyncratic risk" (in which reproductive output is independent across individuals). Populations experiencing idiosyncratic risk enjoy a higher asymptotic growth rate than those experiencing aggregate uncertainty. Therefore, individuals in populations of the former type will have a competitive advantage over individuals in populations of the latter type. Applying this distinction to models of randomly fluctuating environments, Robson *et al *point out that genetic variation among offsprings can serve to reduce aggregate uncertainty, transforming it into a more idiosyncratic form of risk. This transformation underlies the dynamics observed in several models of the role of outcrossing in the evolution of sex. At the idiosyncratic risk, the varying environment favors some or other individuals, and combinative variability subsequently restores the initial diversity of a population. The species in all its complexity remains persistent in time. The species remains unchanged despite the variability of the individuals of which it consists. I think that sexual reproduction succeeded just because the sexual populations are more resistant to evolution than the asexual ones, *i. e. *they are more capable of maintaining their functional integrity and robustness in spite of (or rather owing to) their internal variability.

A clear anti-evolutionary consequence of the cohesion is the fact that the genotypes of the outstanding individuals, those with enhanced competitiveness, are not transmitted to the next generation but, after mating with "ordinary" individuals, scatter and rearrange in new gene combinations, thus preventing the winner from exploiting a success. (The proponents of eugenics and cloning of man are extremely upset by this, but I hope that the *Homo sapiens *species will adequately resist their efforts, so that the mankind will not perish being transformed into *Superhomo asexualis*.) The recombination destroys favorable genotypes more frequently than it creates them [50]. Natural selection does not select outstanding individuals; it pins its hopes on the diversity.

Moreover and above all the theorizing, evolution is really slow. The whole organic world consists of the evolutionary long-livers. Palaeontological evidence for evolutionary stasis is more than convincing, a pattern of punctuated equilibrium is common, and stasis is data, not a theory [92-96]. Recently, it was shown that functional transfer of mitochondrial genes to the nucleus is more common in selfing or clonal plants than in outcrossing plants - the direct illustration of conservative function of sexual reproduction [97].

This conservatism may seem to contradict the data on the evolutionary dynamics of biodiversity during the Phanerozoic, which demonstrates self-acceleration (see *e.g. *[98]). But it is deceptive. The stability of the established species by no means implies its inability to speciate. Rather opposite is true: "Sexual reproduction predominates among organisms mainly because most evolutionary change is concentrated in speciation events, and asexual species cannot speciate in the normal sense" [28]. Robust, long living parents will more readily give birth to numerous (and robust) progeny.

### Species as an individual

A biological species is an individual of higher rank [53-64]. Some people are embarrassed by the absence of common physical skin over the species, but the skins of the constituent organisms are sufficed for the physical delimiting of the species from the environment. Bonding of the intra-species components (individual organisms) is carried out by means of behavior. I suggest using the term "behavioral bond" to designate the interaction between organisms by analogy with ionic, covalent, hydrogen, et cetera bonds. Behavioral bonds provide cohesiveness of groups, species, societies, and ecosystems. Species-specific behavior implies operating of special connections between the individuals, which transform the species into organization with the function of survival. Primarily, these are the connections accountable for the interbreeding and reproductive isolation, which is an equivalent of the common skin. Reproductive isolation is determined by the mutual affinity of organisms. The affinity is not limited by choosing a mating partner; it includes all the intraspecies interactions as distinct from the interspecies ones.

The genetic basis of the integration is a species gene pool. Genetically, a species, akin to an individual organism, is a closed system. Parts of the species (groups, demes) are potentially capable for substantive existence in nature. This capability is analogous to the capability of plants and lower animals to regenerate the whole body from the parts. The absence of the physical skin hampers us to grasp a species as a unity. But it is only the matter of habit. For training of imagination, I would recommend to get acquainted with a bee's colony. It is a clear example of a superorganism made of the individual organisms. It is helpful to imagine the walls of the hive or of the hollow as a colony's skin.

### Species ontogenesis

Having recognized the biological species as an individual, it seems logically justified and heuristically useful to regard the intra-species evolution (microevolution) as species ontogeny. A species has its birth, infancy, adolescence, maturity, aging, and death. Usually, actually always, the terms "multiplication" and "reproduction" are used as exact synonyms. However, these phenomena are quite distinct ontologically and have different biological meaning. The essence of reproduction phenomenon is not increasing the number of organisms. It is rather renewal, replacement the old bodies by the new ones, restoration of the live systems on the basis of genetic information. The essence of multiplication is the biological expansion, spreading out. Reproduction is largely a matter of creative force of the ontogenetic programs; multiplication is largely a matter of contingency.

#### Birth

The biological meaning of speciation differs from that of reproduction of organisms in one important relation. Asexual organisms reproduce their exact copies. Given the very high fidelity of DNA replication in the simple organisms, they reach in this way potential eternity. Sexual organisms cannot reproduce their copies. They exist as constituents of the "species body", akin to the cells of a multi-cell organism. During their reproduction, various gene combinations are tested and selected for vitality and put in the species gene pool. Sexual organisms do not care about their personal eternity. Sexuality does imply this. For a biological species, speciation (species multiplication) also is not a way of its own salvation because the descendant species are not the exact copies of the ancestor. Speaking metaphorically, a species is interested not in speciation (no need to procreate the contestants) but in its own longevity. Speciation is not a built-in stage of the species ontogenetic program. Nevertheless, speciation is possible and it does occur now and then. It happens as a chance event, for example, as a result of geographical or ecological isolation of a small group or just a couple of individuals of different sex [77,99-104].

#### Infancy and adolescence

The Wright's genetic drift inevitably accompanies every speciation event. In essence, the particular (impoverished) gene pool of isolated group is the species bequest. The offsprings of the group will have to mate with each other. This will lead to considerable homozygotization of the arising population. The homozygotic individuals usually have a drastically reduced robustness as a result of losing heterozygosis and baring the recessive (harmful) alleles. The populations they form also loose robustness because of missing polymorphism and loosing genotypic plasticity. This, in addition to a still low size of the incipient species and the specific environment of the new niche, puts the population in a critical situation.

Death of the individuals with bad health and inadequate behavior occurs (severe purifying selection). I think it is not constructive to describe this process as moved by competition of the individual organisms. The competition may occur in some particular situations, but generally and primarily, the organisms die because of their own imperfection. Malthusian interpretation of the fact that not all offsprings survive is not valid. Procreation of the extra progeny is a corollary of the combinatorial character of sexual reproduction, which implies random formation of various genomes including inevitably those of low vitality. This is a moment of the purifying selection. We continue to exaggerate the role of the intraspecies struggle and to underestimate the cooperation and mutual aid, which are fairly evident at every turn. Another reason for the production of extra progeny is the high probability of random death (r-strategy of reproduction).

Yet, on the other hand, the mutations, compensating for the deficiencies, are accumulated. After some historically short period, the isolated population (if it is not extinct, which is quite probable) will become more or less different from the species to which the isolated group had belonged. During this initial period of rapid evolution, new beneficial alleles and those compensating the harmful mutations are accumulated, enriching gene pool of population thus *creating new species robustness*. Robustness is a complex phenomenon, embracing the capability of individual organisms to perform successful development in the background of mutational and environmental perturbations and genotypic plasticity of populations [25]. During this short period of the species life, new form is created, with its special genetics, biochemistry, physiology, morphology, and behavior.

The crucial moment for the emerging species occurs when, for one or another reason, a contact of the isolated population with the ancestor species is restored. If during the time of isolation, a reproductive barrier against the ancestor species was put up, the new species will be fixed and the creation of unique coadapted gene pool [100] becomes possible. If the reproductive barrier was not put up, the new species will not come into life, being dissolved in the maternal species (such events are discerned sometimes in the fossil record [105]).

Reproductive isolation is an important species homeostatic mechanism. Sexual reproduction was necessary because a higher organism is unable to cope with the mutation flood by acting alone. On the other hand, promiscuous crosses would be lethal for the species because of its dissipation. There are many different isolating mechanisms: ethological, anatomical obstacles for mating, sterility or low vitality of the hybrids, and inability of the fertilization. Most important is the ethological (behavioral) mechanisms of isolation. All animals have rather complex systems for identification of the individuals of the same species (modes of courting, species-specific signaling and so on). A noticeable example of such psychological incompatibility of two species presents the wolf and the dog. Physiologically they are completely compatible, hybrids are fully vital and fertile, but they hate each other, and this hate helps them to maintain themselves as separate species. One more example: "Oh how similar to us is the hideous beast simian," said Karl Linné. (It was citation from Ennius Quintus: "Simia quam similis turpissima bestia nobis!") Why is a monkey so repulsive to us? A horse and a lion are beautiful, but a monkey is ugly. Long ago, during the formation of *Homo sapiens*, this aversion to our ancestor saved us from dissolving in it and made our independent evolution possible.

The isolation mechanisms have an anti-entropic, organizing function. They are analogous to counteraction to diffusion. Reproductive isolation is necessary step in creating an individual of a higher rank - biological species: the system becomes genetically closed. This is a "skin" of the superorganism. Ethological isolation mechanisms are especially suitable to demonstrate how anti-entropic behavior is possible without a special expense of energy. We probably must admit that quite a good price was paid for the individual's present-day capability of correctly choosing a mating partner: infertility of all the ancestor individuals that were devoid of such capability. But when analyzing only the present-day events, we have to ascertain negentropic behavior. The well-known ethological phenomena of sexual and filial imprinting (see *e.g. *[106]) are actually the teleonomic mechanisms for the maintenance of the reproductive isolation and behavioral integration of the species.

#### Maturity

Later, as the size of the population increases and its gene pool gets better coadapted, the probability of the successful fixation of the novelties becomes lesser, and the rate of the visible evolution moves to zero. The species reaches stasis. The stasis does not imply complete cessation of the genetic changes. The life of a species is never-ending struggle against mutational and environmental perturbations. A species may reach adequate adaptation to the varying environment by creating intra-species genotypic diversity, enabling it to support optimal phenotypic configurations without committing irreversible evolutionary steps, but it cannot cope with mutation flow once and for all. Persistence of the species is not like passive persistence of a granite rock, it is permanent restructuring, never-ending routine repair of the species genetic pool. And purifying selection works permanently to the same goal, removing organisms and populations of low vitality. Of course, in nature, there is no selection other than selection for survival. In the case of success, it leads automatically to robustness of individual development and genotypic plasticity of populations (*i.e*. to the halt of visible evolution).

#### Aging and extinction

We know well that species die. Moreover, the great majority of them did die [107]. There are a lot of external causes of extinction. But are species potentially (*i.e*. in a fully favorable environment) immortal? For simple organisms, such as prokaryotes, we may probably say: "yes, they are". They are capable of reproducing themselves with very high fidelity, suffering about one change in DNA sequence per 300 progeny cells [65]. For higher organisms, the answer is not so evident. The apparent evolutionary stasis of higher species is a multifaceted phenomenon. We know that they may remain phenotypically unchanged during millions of years, but we also know that genotypically they continuously change, and the mutation flow cannot be stopped or at least slowed down. New mutations infiltrate the gene pool, and these are constantly neutralized by compensatory genetic changes. Intuitively, this "financial pyramid" of evolution does not seem to work endlessly. It may possibly crash down when the species capacity to resist entropy pressure becomes inadequate. In the ruins, new small group may undertake an attempt to create another variant of the species, which may happen to be more perfect than its predecessor and live longer. But this would be another story. The descendant species may not be regarded as a conqueror of the progenitor species. For example, G. G. Simpson [108] construed direct interspecies competition as rarely the cause of extinction of species. He thought replacement of one species by another relative species was largely passive: "...the usual sequence is for one dominant group to die out, leaving the zone empty, before the other group becomes abundant..."

Thus, it is important to distinguish two major periods in a species history: *coming into being *and *stable existence*. The coming of a species into being, its birth and maturation, is a phase of the generative evolution. It is a period of ephemeral and risky existence of the young species that does not leave any traces in the evolutionary chronicle. It is the "petiole", which P. Teilhard de Chardin spoke about to explain the absence of the intermediate forms in the present-day biosphere and the interruptions in the palaeontological chronicle [109]. Only those are retained that reach a certain level of perfection, a capability of surviving without visible altering. Just these two periods of evolution are implied in the conception of the punctuated equilibrium [110]. Thus, the microevolution is species ontogeny, whereas its content is creation of new robustness.

There is a principal difference between the ontogeny of multicellular organism and the ontogeny of multiorganismic species. The first is a predetermined, goal-directed, governed process, representing an empirical embodiment of the ontogenetic plan [25]. It is reproduction of the body on the basis of genetic information. The second is an open-ended, *creative *process moved by entropy. It is restricted historically (by the gene pool of the founder) and environmentally. The major attractor for the organism's ontogeny is the mature form; the major attractor for the species ontogeny is stasis. An English word "creation" is commonly used in a rather broad sense as the opposite to destruction. In the context of the present paper, this word is mainly used as a synonym of the "artistic creation," which includes fantasy (variation) and taste (selection). Evolution creates like an artist, not like an engineer. Empirical search, trial and error plays an essential role in the evolutionary process.

We see two opposite trends in the biosphere. Mutagenesis and an inability of organisms and species for reliable self-reproduction are the manifestations of the never-ending attempts of the chaos intrusion into the ordered and organized structures and processes of life. But there goes the never-ending improvement of the systems that resist the chaos. Both of these trends increase in time. An ever complicating biosphere as a whole and the appearance of ever more complex and highly organized organisms and systems increase their vulnerability to entropy pressure, their thermodynamic tension. But in parallel, ever more complex and diverse, ever more perfect mechanisms of resistance to chaos at various levels, from the molecular to the spiritual one, lead nature farther and farther from the thermodynamic equilibrium. It is a pity that the generative side of evolution (inevitable concession to chaos) is usually taken as the major (and positive) contents of evolution, whereas the achievements of evolution in the ways of resistance to chaos (labeled as stasis, stagnation, evolutionary failure, evolutionary blind alley) are looked upon at least disappointedly.

## About levels of selection

Speaking about species as individuals, it is impossible to avoid the discussion of the problem of units and levels of selection and evolution. The question - does natural selection operate on genes, individual organisms, groups, or species - is an "accursed problem" of the evolutionary thinking. Discussion of this problem occupies a great part of the publications in the last fifty years (see [1,60,111-130] for the range of the incompatible opinions). To a great extent, this incompatibility reflects the difference between the reductionistic and holistic philosophy of the participants. I have a funny idea that this difference is rooted in the genetic level, so it may be overcome only after long evolution.

It is getting popular to speak about multi-level selection. That is the progress in comparison to the adamant "gene-centered view". I would like, however, to stress that a complex entity like a species cannot stably exist unless it is *an organization, i.e*. hierarchically structured system with the function of survival. This implies constraints on the behavior of the system's constituents. Selection at the lower level will disrupt integration at the higher [124]. Hierarchy in biology implies a functional predestination of the parts for the sake of the whole. In accord with my holistic attitude, I dare to make one more step and suggest that there is no such thing as synchronic (see [130]) multi-level selection. Natural selection just cannot select anything that is not a *unit of reproduction *(see [128]).

In the case of sexual organisms, the minimal unit of reproduction is generation of population or group. As is stated in Wikipedia "Selection at the level of the organism can be described as Darwinism, and is well understood and considered common." It is really well understood but only in the case of asexual lineages, in which individual organism is a unit of reproduction. Sexual organisms have lost the capability of self-reproduction. They are temporary, renewable, perpetually varying constituents of the species. They place their genes into the common gene pool, hereby demonstrating the hundred-per-cent altruism and placing evolutionists in an awkward position: individual organisms, commonly construed as quintessential units of Darwinian evolution, cannot be selected as such. Thus, I say in favor of group selection as the only meaningful level of selection for the obligatory sexual organisms. Let me cite a statement made by one of the most adamant reductionists: "One feature is common to many of the transitions: entities that were capable of independent replication before the transition can replicate only as part of a larger whole after it" [1]. Given that the higher level selection does operate, the selection at the lower levels, if allowed, can produce nothing but casualties like a parasitic DNA or a malignant cell. Ultraselfish genes or other parts of the whole are factual (not metaphorical) parasites with net harmful effect on the host. Are we allowed to think that progressive evolution causally related to the harmful mutations even if they are prerequisites for their overcoming by subsequent evolution? Even if it is really so, we should strictly distinguish between the problems and their solutions. Ultraselfish genes, along with other parasites and harmful mutations, are representatives of the destructive force of nature. Evolution in action is unending struggle against this force. The most productive way of this struggle is cooperation.

### Selfish gene

I cannot avoid saying a couple of words about the selection of genes for the good of the genes [114]. Neo-Darwinist comprehension of a gene looks a bit mystical to me - such an almighty little one. Probably, this demonization of the gene results from the direct transfer of our vulgar comprehension of the relationships within human societies to biological systems. The expressions like "protein P53 controls..." or even "nitrogen oxide controls..." are laboratory jargon unacceptable in theoretical formulations. Nitrogen oxide is too stupid to control anything. That who controls must be higher organized entity than those who are controlled. A part cannot control the whole. When we say that gold rules the world, it is not more than a figure of speech. Gold has no power in the world of plants and animals. Signals, releasing factors, orders, and laws acquire causal character and rationality only in the context of the whole. Therefore, we can say that only a system makes decision. Genes are a passive memory of the cell, its notebook, not its mind and will. To ascribe the ruling function to the genes is the same as to ascribe the knack to drive a car to a road chart, not to a driver.

One may think that a very long time ago in the prior-to-cell world, macromolecular replicators (the ancestors of genes) were substantive entities and evolutionary individuals. But it is only possible to credit the modern genes with such a status because of great respect to Richard Dawkins. I would like to "But truth is more valuable for me". A gene is frequently taken as an ideal replicator. But it is not a replicator at all. It is replicated. It is a replica or a template. Genes are reproduced by the cell, just like the other cell constituents: RNAs, polypeptides, organelles.

One may make an objection: What about a general in command of an army? But the situation may not be as simple as it looks on the face of it: an order is absolutely nothing if the army is not disciplined. "The acting entity" is the whole, which is general plus army that is ready to fight, to win or die. The holistic view should not be oversimplified as absolute superiority of the whole over its components. I agree with the statement that Voltaire is cleverer than France. Moreover, any Frenchman is cleverer than France. The ability to abstract thinking is a property of human individuals. Human societies, including the Academy of sciences, are devoid of this ability. A biological species is an organization of a higher rank as compared to an organism. It consists of organisms. It looks evident, however, that an individual organism in many respects is a more organized system than a biological species. Let me present an example of a very simple organization of a higher rank than an organism: a troika (three horses harnessed abreast). It is certainly an organization, the organization with the function of transferring passengers. It is a cohesive whole. It includes three horses, a carriage, a harness, and a coachman. But the horses and especially the coachman constitute a much higher organized system than the troika. The superiority of the troika relates only to its ability to transfer people. If we mean just this function, then we must admit the dominance of the whole over its components and ignore the complexity and perfection of the components. The function of the biological species is survival. In this respect, the species is more perfect than the organism, and moreover, it makes the survival of the organisms themselves possible as a class of entities. But the matter looks true only from the evolutionary point of view. In other aspects, the organism may by far excel the species, like Voltaire as a thinker excels France. I think that even a cell is more sophisticated and perfect in some respects than a multi-cellular organism, though the function of survival belongs to the organism, not to its constituents.

The holistic view implies that the unit of selection is always and only a substantive entity. Of course, its evolution means changing of the constituent entities, but the natural selection retains only those changes that are beneficial (or at least harmless) for survival of the host-entity. The survival is provided by the co-coordinated activity of the constituent entities. This co-ordination is an emergent quality of the substantive entity that does not belong to any of its components. When the cell came into being, the substantive existence of genes came to the end. Genes have sacrificed their freedom for the security and reliability. And they made a wise choice. What do the genes need for an eternal life? They need a high fidelity of their replication. And they have it: less than 10^-10 ^errors per base pair! Where are the free, self-sufficient ancestors of the genes now? We are not even sure that they really existed. As for the present-day genes, they have no other care than the care about the welfare of the cell, the organism, and the species as a whole. The frequency of harmful mutations exceeds that of beneficial ones by four to five [131], or even six [132] orders of magnitude. So, evolutionary initiative of a gene usually comes to a bad end. Do genes themselves evolve? They do. They underwent the mutation pressure and accumulate the neutral changes and those that make them suitable for a certain genome, organism, and species.

It gets increasingly clear that the concept of a selfish gene is not based on real premises. The linkage "one gene - one trait - one selection vector" is not observed: one gene may affect several traits, and most traits depend on many genes. Phenotypic expression of an allele depends on the genetic context. The notorious reversible replacement of the white and black forms of the peppered moths is a rare exception. You cannot select a trait. For example, you cannot select velocity of run. You must select a swift-footed animal. But to be swift-footed means to have good muscles, bones, general design of the body, blood-system, lungs, heart, nerves, hormones, coordination of movements, motivation to run, and so on. In other words, you need the whole organism adjusted to running. Thousands of genes must be involved in this selection. And it is not the whole story. Changing thousands of genes may affect the functional activity of thousands of other traits important for survival. But a sexual organism cannot produce its copy. Hence, you may only select a population of swift-footed animals.

We are not adjusted to thinking within a holistic paradigm. Our analytical brains fill comforted hearing that gene alleles are selected (delusion of the profound understanding). Of course, changing of a whole does imply changing of its constituents. There is no other way of changing the whole. But is this a sensible knowledge? Let me give a clarifying example. Evolution is a creative process akin to other creative processes. Formally, we may state that when a poet writes verse, he selects letters and words. But this correct statement is vapid. Actually, he selects something that the letters and the words do not contain. And natural selection selects something that the genes and other parts of the unit of selection do not contain. The products of biological evolution - biological entities - are empirical godsends fixed by the natural selection for their ability to persist. They cannot be deduced via reductionistic logics. And their evolution may be comprehended only retrospectively. A good gene and good individual are not selfish ones but those blending appropriately with the species gene pool and population to sustain their vitality.

What is really tested during species ontogeny then? And what is selected? It is the quality of the gene pool as a whole, its ability to sustain the population survival, its robustness. The most important element of the population robustness is the genotypic plasticity. Of course, for the selection to be possible, the general population must be structuralized, *i.e. *contain more or less reproductively isolated groups [133]. Such partial isolation must inevitably arise because of the geographical and behavioral factors. For example, sexual and filial imprinting may be a common mechanism for the limitation of panmixia, continuously creating local subpopulations. On the other hand, various mechanisms preventing inbreeding provide a reasonable genetic diversity of the sub-population. Unlucky groups may be replaced by more perfect ones. This process must be intensive during the early stages of the species ontogenesis. Opponents of the group selection reject it as a too slow process: "lower-level selection easily trumped higher-level selection". First, the group selection may be rapid enough: a generation of population (the unit of reproduction) is of the same longevity as an individual organism; second (and uppermost), the lower-level selection in itself is the destructive side of the overall process. If it is not trumped by the higher-level selection, the species simply will not go through. Most probably, the overwhelming majority of speciation events end with an abortion. And again, competition between the emerging sub-populations may play little role in their extinctions: they parish because of their internal imperfection, *e.g*. because of the prevailing of selfish behavior of the constituent entities.

There are global biological phenomena that are difficult to explain from the individual-centered position but appear just natural from the species-centered view. One is the origin of altruistic behavior of individuals. The known neodarwinistic explanations (see [134] for a recent review) are in fact may be (and should be) construed as group-selection. In addition, they in fact imply a monogenic determination of altruism that does not look probable for such a sophisticated trait. I would like to stress the following. It is quite evident that an individual organism works for the survival of the species *primarily *by providing its own survival. A body of the species is composed of mortal organisms. The lasting existence of the species depends on its ability to renew its body via production of organisms. This process is analogous to the renewal of the tissues of organisms, *e.g. *leaves of the tree. One may discern fighting even between the leaves: they shade each other and fight literary for the place under the sun. But we should take into account that all this competition has a definite altruistic meaning - the most efficient feeding of the tree. And even after death and decay, the leaves continue to feed the tree.

The expedient behavior of an individual must be beneficial both for the organism itself and for the species. Most probably, the altruistic/egoistic phenotype of a given individual is determined by numerous genes, and the population is characterized by a broad continuum of individuals, from the "pure altruists" to the "pure egoists". This distribution is the "species trait". Owing to the gene pool shuffling, it is totally transmitted to the next generation, even if the extreme altruists do not produce their own offspring while extreme egoists have too little concern for their offspring. During evolution, the form of this distribution is optimized for the species survival. Of course, it is species-specific and must be coordinated with the *general strategy of species survival*.

One more phenomenon, inconceivable from the individual-centered view, is phenoptosis, the programmed death of organisms (see *e.g. *[135]). It occurs in the most expressive form in salmon: death of the adult individuals after spawning. It looks probable that the phenomenon of aging is just a slurred form of the phenoptosis. The different longevity of the individual life is also manifestation of the same phenomenon. Why does a mouse live only two years, while a man lives up to hundred years? The answer is: such is the general strategy of the species survival. The answer is too general of course but it is correct.

Especially interesting is the regulation of fecundity. The problem was brilliantly presented and reasonably resolved by V. C. Wynne-Edwards [111] but it was rejected violently as not fitting the neodarwinistic concept. Why does the cod lay about 5 million eggs, while the elephant female produces a calf every four years? The general tendency in progressive evolution is diminishing the fecundity. It is funny that the relative fecundity is a key parameter of population genetics reflecting evolutionary success of an allele or an individual organism, assuming, therefore, that the more the better. The very idea of natural selection was based on the observation that organisms produce more offspring than can ever survive. Organisms therefore compete with each other for the limited resources. The fittest (*i.e*. more fertile) survive. This competition was construed as a moving force of evolution leading to continuous perfecting of the biological entities. But there is a question: why is the offspring produced in a quantity that is beyond the life-support resources? Some say: "For evolution", thus unconsciously (or consciously?) taking up Lamarckian "innate drive for perfection". I think it is for unfailing reproduction, *i.e. *for survival of the lineage. Extra copies are made for two main reasons: to compensate for the poor fidelity of reproduction (internal factors) and to compensate for random death of the organisms (external factors). Competition between asexual lineages and between different species looks natural but the competition between organisms of the same species is akin to the competition between, *e.g.*, hepatocytes and neurones. I do not discard competition and struggle, but their biological meaning should be reconsidered: they are the instruments, means, and ways for creating, fine-tuning and maintaining cooperation. The emphasis on the fierce struggle just creates the problems in the theory that do not exist in reality.

## Competing interests

The authors declare that they have no competing interests.

## Reviewers' reports

Reviewer 1

Pierre Antoine Pontarotti, Directeur de Recherche CNRS, UMR 6632 Université de Aix Marseille/CNRS, France

This article proposes hypothesis that could explained "why" sexual reproduction occurred and its role in the evolution of the multicellular species

General comments

I consider that if a scientist proposes a hypothesis he should clearly depict it and present significant clue to test this hypothesis. In my mind, I think that the article should be rework in these two directions to be understood by the community.

***Authors' response: ****It is possible, of course, to consider my paper as a hypothesis, though I would prefer to denote it as a discussion, the discussion of the problems related to sexual reproduction. Sexual reproduction is really a puzzling phenomenon because it looks unnecessarily complex, costly and risky. So there is no wonder that many (not only me) ask "why". There must be a very serious reason for the ubiquity of sex. My answer to this question is the heading of this paper: "The biological species is the only possible form of existence for higher organisms". In other words, I suppose that asexual forms of such a level of organization cannot survive in the long run. The real asexuality is limited to the simplest forms of life, mainly, to the prokaryotic organisms and to a few rather simple eukaryotes like Bdelloid rotifers. Asexual and sexual organisms use different strategies of survival. Strange as it may seem, it is not easy to define what survival is. The standard antonym to survival is death. But what about changes? To what extent an entity may change while remaining "the same thing"? It depends. It depends on the definition of "the entity". A human being changes from zygote to old man/woman. If we speak about the zygote, it disappears after the first division; if we speak about the individual organism, it disappears after death. An individual bacterium disappears after division. Successive divisions of cells provide survival of the lineage. Disappearance of the lineage is extinction (death). But what about transformation into another lineage (via phyletic gradualism)? Does it mean the disappearance of the ancestor lineage? Yes, it does. I find it useful to define survival as a retaining self-identity. In the evolutionary context, to exist means to exist for a long time. There are two ways of ceasing existence: to die or to be transformed into another lineage. Hence, to survive means to resist both death and evolution; the forms, that are resistant not only to decay but also to evolution, are selected. As far as I know, the resistance to evolution was never overtly construed as a positive selectable trait, though purifying selection and stabilizing selection imply this covertly. Asexual lineages, being either unicellular organisms or organisms with a small number of germ-line cell divisions, survive due to the very high fidelity of DNA replication *[66], *which is enough for the reliable self-reproduction. But the replication with high fidelity is a costly process. It looks probable that the attained fidelity of DNA replication per base pair per cell division is close to the maximum *[67,68]. *Consequently, the per-generation rate of mutation in organisms with a large genome and a large number of germ-line cell divisions is very high, up to three orders of magnitude higher than, for example, in yeast *[71-73]. *I.e., genomes of higher organisms are not reproduced with high fidelity. The mutational deluge menaces to destroy both homeostatic mechanisms of the organisms and the evolutionary stability of the species. But we know that species stably reproduce themselves during dozens of millions years remaining the same, at least in the opinion of paleontologists. In this paper I am trying to understand how it is possible."*

*One more general remark which, I hope may facilitate accepting of the paper. On one hand, the theory of evolution is a province of science. But on the other hand, it is a world outlook, the essential component of the modern philosophy. In the present paper, devoted to rather general problem of species and sexual reproduction, I cannot help seeing the problem in the general context of science and philosophy. This partly explains a bit declarative and speculative stile of the paper*.

*Reviewer 1*

Specific comments

You write "simple organism like bacteria". How do you define simplicity and why do you think that bacteria are simple organisms? (I know that it is written in a repetitive manner but we need some explanations here)

***Authors' response: ****I use the word "simple" in its common meaning, as opposite to "complex". In the present context, I mean single-cellularity (hence, simple development) and relatively short DNA (prerequisite for the precise copying) of prokaryotes. Of course, bacteria are very complex and highly organized systems in themselves. Still they are the simplest entities among extant organisms. They are even simpler than the eukaryotic unicellular organisms. (Viruses are simpler than bacteria but they are not substantive organisms)*.

*Reviewer 1*

You write: "The core outcome of the transition to sexual reproduction was the creation of multi organism entity". How do you deal with the fact that some unicellular organisms have also sexual reproduction (for example paramecium, yeast...?)

***Authors' response: ****In the present paper, I deliberately limited the discussion by the instance of the obligatory sexual reproduction where its biological meaning is expounded in the most obvious form (at least for me). I think that regular sexual reproduction, even facultative, always creates biological species because of emergence of the common gene pool. However, the species may have cohesiveness of different degree. Those with obligatory sex are maximally cohesive*.

*Reviewer 1*

Page 2 lane 5: You write: "This megagenome with its billions of chromosome enables the species to create huge genetic diversity, which is not accessible for the asexual species"

Please, specify that HGT is also present in asexual species (and in some cases in sexual species) and therefore allow them to create genetic diversity. Please add a comment on that point.

***Authors' response: ****This peace is within ABSTRACT. I add the required comment in the text*.

*Reviewer 1*

"Malthusian..." Please provide a reference

***Authors' response: ****This is also in ABSTRACT. I could give the reference, e.g. *[136]*(which I did not read, of course, and, I think, nobody will). I mean here the well-known Malthus's idea of unlimited exponential growth of population leading to the competition for resources and struggle for existence. I give another explanation for the production of the extra progeny. The procreation of extra progeny really needs explanation*.

*Reviewer 1*

I you write "I declare..." I do not think that such a word can be used in a scientific paper. You should write instead that your analyses allow you to put forward the following hypothesis .....

***Authors' response: ****Done*.

*Reviewer 1*

What do you mean by "the intermediate configuration being volatile?"

***Authors' response: ****This is just continuation of the previous statement that the Universe consists of entities, i.e. it is not just smooth continuum of matter. For example, there is no atoms intermediate between hydrogen and helium: they are impossible because they are unstable (volatile)*.

*Reviewer 1*

"biological evolution cannot retain everything that randomly emerges." This is statement please explain better and give references.

***Authors' response: ****For me, this statement is self-evident. There is no wonder in inability to live. Only ability to live needs explanation. In particular, we know that interspecies hybrids are of low vitality or evolutionary unstable even if they can arise. I wanted to say that the absence of smooth continuum of living forms is an apparent (seeming) problem that needs no special explanation. Only extremely rare combinations of atoms and molecules correspond to living forms*.

*Reviewer 1*

You state that non multicellular organism cannot be organized in species (in different places in the article). I convince that the word species could have different meanings. You should clearly explain the concept (or at least your own concept of species) and explain why the non multicellular species cannot fit your definition. Any way for example bacteria (even with metazoan) is capable of forming highly "complex" community.

***Authors' response: ****No, I did not state that single-cell organisms cannot be organized in species. Having sexual reproduction and organization in species just the same thing. I am aware about extensive discussion of species concept and related things (what is individual, what is organism and superorganism, what is Darwinian individual). In the present paper, I tried to evade this discussion (which is a bit scholastic). Mayr's definition of biological species *[13]*looks quite enough for me: "Species are groups of interbreeding natural populations that are reproductively isolated from other such groups." In revised copy, I cite this definition. So far, I am not ready to discuss here the very interesting phenomenon of "highly 'complex' communities" of a higher than species entities (ecosystems). At first glance, they are organizations of a higher than species rank though with weak (if any) genetic cohesiveness. Of course, they are not biological species*.

Page 5

*Reviewer 1*

Lane 6: please provide reference

***Authors' response: ****You mean "The prosperity of the whole is the vector of selection for the constituent entities?" I doubt that I can or should find the required reference. It is my own logical conclusion. Moreover, it is my major conclusion. And it is self-evident. Sexual individuals can exist only as parts of the whole so the survival of the whole is a prerequisite for their own existence. They must be adjusted to the whole. They must be selected for the good of species*.

*Reviewer 1*

Lane 20 you write "means to persist" Please provide a reference

***Authors' response: ****You mean "survive means to persist". What reference should I provide? The words survive and persist are synonyms in English*.

*Reviewer 1*

In fact all the lineage dies (coalescence) and a species can be seen as a given lineage

***Authors' response: ****Sorry, I did not catch the question (or statement)*.

*Reviewer 1*

"Are far from the maximum entropy ". Please, develop and explain what entropy is and how entropy could be related to biological work (even if it is evident for you).

***Authors' response: ****Organisms and species are organizations. Organized systems are far from the maximum entropy by the definition. They are extremely improbable. This paper is not about the thermodynamics of sexual reproduction (I even cannot imagine what it could look like). And it is not about the transformation of energy. Throughout this paper, I use the word "entropy" in generally taken sense as an indication of disorder or disorganization. Entropy and Second Law are notions related not only to transformation of energy. Rather they are universal characteristics of systems of any rank. Entropy is a convenient term for designation of general propensity of the real systems to move to more probable state, to chaos. Brooks and Wiley *[6]*equate evolution and entropy. This is only partially true. Mutation is entropy-moved process indeed, but natural selection selects and maintains variants possessing vital capacity that resist entropy pressure and further evolution owing to their homeostatic mechanisms, their expedient behavior. One of the principal propositions of the present paper is the suggestion that major menace for living entities is not rivals or enemies but their own imperfection. What is the entropy growth? It is errors of replication, errors of transcription, mistranslation and so on. Aging and any wearing out is an entropy growth. There are many causes of death but only growth of entropy is ever-acting and inevitable one*.

*"An everyday example of entropy can be seen in a deck of cards. A deck ordered by suit and number will tend to progress towards a randomly arranged deck upon ..shuffling, because the latter system has more possible states than the former. Furthermore, this process is thermodynamically ****irreversible****; restoring the deck to its ordered state requires the application of work. The recovery of the ordered deck via the random process of shuffling is highly unlikely because the random deck has a much higher entropy" (Wikipedia).There is one important omission in this note: application of energy would not be enough for the restoring the deck; it is necessary to know what and how to do this. Energy in itself is chaotizing factor. Natural selection, this 'blind watchmaker' "knows" what to do. It selects low-probable (i.e. low-entropy) vital variants. (See also addition in P. 6 of the revised version)*

*Reviewer 1*

Page 7 Lane 3, Please explain

***Authors' response: ****Do you mean "We are probably allowed to state that the evolvability arises as a byproduct of the mechanisms aimed at conserving genetic information"? I may remove this phrase from the text (if you do not like it) since it is not essential for the problem discussed (it is aimed against idea of evolvability as selectable trait). I mean here that for evolution to be possible, multiple offspring is necessary. But multiple offspring are produced not "for evolution" but "for survival"*.

*Reviewer 1*

Lane 8, from: "I would like to explain ...survival" I do not understand how this paragraph is related to the present discussion; Please, clarify.

***Authors' response: ****I compare reproduction of asexual and sexual organisms and describe the corresponding inheritable units: a cell (not just DNA) in case of asexuality and a generation of population (not just individual organism) in case of sexuality. I regard this description essential for the holistic view*.

*Reviewer 1*

"Natural selection is traditionally construed as selection of novelties". I do not agree with this statement, most evolutionary biologists think that natural selection correspond also to counter selection. If you think that scientists mix the two concepts (natural selection and apparition of novelties) please provide examples from the literature)

***Authors' response: ****Yes, probably I was wrong (if to mean the evolutionary biologists). I remove this statement. But see below*.

*Reviewer 1*

"The function of natural selection is largely conservative:" please provide a reference

***Authors' response: ****First of all, the very logics of natural selection - "survival of those who survive" - is primarily conservative: reproduction of previous generation. I mean just this. Another thing, that this conservative reproduction is hard to be named "selection". Rather it looks as absence of selection. Still it is selection and the major one. It is selection of vital forms: stabilizing selection and purifying selection. This comprehension of selection was clearly expressed by M. Kimura [e.g. *[12]*] and long ago by I.I. Schmalhauzen *[11]).

*Reviewer 1*

How do you define higher organisms? Again in this paragraph, we still have the problem of the definition of species. Please, see my comments above.

***Authors' response: ****Frankly, I was sure that all the biologists uniformly understand the rather loose term "higher organism". I am aware about modern all the rage to neglect the scale of rank. Some even declare that bacteria are much higher than the animals, those "guts with gonads". Still the traditional vision of biosphere as the scale of rank makes sense, I think. "Higher" does not necessary mean "better" or "more perfect". One unbiased criterion of the level of organization is complexity: the whole is higher than its parts. Another criterion (not so unambiguous) is the evolutionary origin: mammalian were originated from reptilian, Homo was originated from Australopithecus. Such presentation is convenient even if for the purpose of taxonomy. In context of this paper, it is enough to define the higher organisms as organisms with large DNA and complex development. Throughout this paper I mean primarily obligatory sexual organisms*.

*Reviewer 1*

Note that communities of bacteria, archea are higher orders than individual. I really would like to have a comment on this.

***Authors' response: ****Of course they are*.

*Reviewer 1*

Lane 18

Please note that for example bacteria evolve also, by gene substitutions, HGT, gene loss...

see for example [137].

***Authors' response: ****Of course, stability of the species is not absolute. It is not easy to avoid evolution. In this assay I am trying to understand how species resist evolution, how they manage not to evolve*.

*Reviewer 1*

Page 9

Lane 2: Why do you think that evolution is progressive? Please give arguments.

***Authors' response: ****I do not speak that every evolution is progressive. I also do not equate "progressive" and "more perfect". But I cannot agree that there is no progressive evolution at all. Under the progressive evolution I mean transition to a higher order entity: elementary particle → atom → molecule → macromolecule → prokaryotic cell → eukaryotic cell → multicellular organism → biological species → ecosystem. If we do not see "progressive evolution" on the molecular level, it does not imply its absence in general. Absence of progressive evolution on the level of alphabet does not prove absence of any progress in the literature. Not genes but their particular combinations create living entities and help them to persist in time*.

*Reviewer 1*

Lane 5: How do you deal with hybrid species (allopolyploid species for example?)

***Authors' response: ****I was not especially involved in this phenomenon. This breakage through reproductive isolation may be important for speciation, I think*.

*Reviewer 1*

Lane 8: I do not think that in science we can ask why? The only question that one can ask is how?

***Authors' response: ****You are too pedantic (or vigilant). No science could appear without astonishment. "Why" goes first, "How" goes then*.

*Reviewer 1*

What do you mean by nature? In your manuscript, nature looks like an entity?

***Authors' response: ****It is a metaphor. I accept your suggestion, though I like metaphors. And what is wrong with the using nature as entity? It is entity. Not person, of course*.

*Reviewer 1*

I would change the wording by: "what is the evolutionary process that gives rise to the complex way of multiplication "

***Authors' response: ****Accepted*

*Reviewer 1*

Still in this paragraph, it is not true that each asexual transmits its entire gene to the offspring's, see above.

***Authors' response: ****I mean here rule, not special cases. "Asexual individual transmits all of its genes to the offspring (not half)". Of course, they may mutate, acquire or loose plasmid. But these special events are not defining characteristics of asexual reproduction*.

*Reviewer 1*

Please note that the environment is not constant, therefore even if a species is perfectly adapted in a specific environment, it would not be strictly the same case in other environment (please have a look on the SJ Gould book: Wonderful life [138].

***Authors' response: ****In this paper I speak about evolution of resistance to evolution and try to show that sexuality helps a species to retain its self-identity despite mutation and environmental perturbations*.

*Reviewer 1*

"Since most of the mutations are harmful..." First table brings to mind that different kinds of mutations exist: punctual deletion recombination (leading to gene loss end exon shuffling...). In the case of punctual mutations many of the mutations are neutral: for example synonymous mutation. In any cases, if you think that most of the mutations are harmful, you should provide references.

***Authors' response: ****Sorry, it was my overlook. I know that most point mutations are phenotypically and selectively neutral or nearly neutral (fortunately). I have this corrected*.

*Reviewer 1*

"A clear anti evolutionary consequence..." Please note that positive selection exists also in sexual species, a given allele under positive selection will take longer to be fixed in sexual species than in asexual one, but it can be fixed.

***Authors' response: ****I agree. I do not say that sexual organisms do not evolve. But see next paragraph*.

*Reviewer 1*

"Being captivated with the idea of progressive evolution": who believes that evolution is progressive? If you have scientific references stating that, please provide them.

***Authors' response: ****I have removed this irritating phrase*.

Reviewer 1

What do you mean by "lower animal"?

***Authors' response: ****For example hydra and flatworms*.

*Reviewer 1*

"Asexual organisms reproduce their exact copies..." This is not true, see above, many genetic events occur such as HGT, gene loss ...

***Authors' response: ****I mean that the gene shuffling is not an obligatory step during reproduction of asexual organisms. HGT may play essential role in evolution of prokaryotic world. But I mean here just reproduction, not evolution. For reproduction HGT is not necessary. Just like mutations and other genome perturbation are not obligatory elements of reproduction. They are rare mistakes. Prokaryotes are able to reproduce themselves with very high fidelity. Sexual organisms cannot reproduce their copy*.

*Reviewer 1*

Please note that the monkeys are not our ancestor, we share a common ancestor, and this ancestor gave rise to different species including human.

***Authors' response: ****According to my university's diploma, I am zoologist. So I know well that Homo is one of the monkeys. Excuse me my joking. I did not say that monkey (modern monkey) is our ancestor. But I think that our common ancestor had "monkey-like" features that were repulsive for our "human-like" progenitor and they are still remaining so for us*.

*Reviewer 1*

"the rate of the visible evolution asymptotically moves to zero": please provide a reference

***Authors' response: ****I remove the non-circumspect epithet "asymptotically". I just mean the evolutionary stasis. Palaeontological evidence for evolutionary stasis is convincing *[92-96,139,140]

*Reviewer 1*

" For higher organisms the answer is not so evident". Please note that mutation rate has been published many times in the case of metazoans.

***Authors' response: ****I am aware about these data and cite them here*.

*Reviewer 1*

"This means that the species are more longevous that the genes." How do you deal with transspecific polymorphisms, allele sorting...?

***Authors' response: ****I remove the phrase about longevity of genes because it over-simplifies the real situation and it is not essential for the problem of species longevity. As for the trans-specific polymorphism and allele sorting, I would prefer not to discuss these phenomena here. One of my reviewers already reproached me (justly) in attempt to say too much in one paper*.

*Reviewer 1*

"Evolution creates like an artist". Do you really think that evolution could be regarded as an entity?

***Authors' response: ****Again it is metaphor. It would be inelegant to compare an artist with a process*.

*Reviewer 1*

I think that you should publish your manuscript with my comments as they are all the best.

*Reviewer 2*

Michael T. Ghiselin, California Academy of Sciences, USA

I am not in a position to write a formal review, especially given the arrangements. However, I would like you to have my comments on the paper. Your manuscript deals with a wide range of topics and I think that you are trying to say too much in a single paper. You should either make it more focused or write a book.

***Authors' response: ****I really made my best to concentrate my discussion on one question (why sex) and give one answer (to survive) but probably, I failed and did not manage to evade discussion of numerous closely related topics*.

*Reviewer 2*

I am familiar with much of the literature on sex, but have not tried to read everything of significance on that topic. I am also familiar with holistic thinking, and recognize the influence of Russian authors on your views. I read Russian so I know some of the literature in your language. In 1974 I published a book entitled "The Economy of Nature and the Evolution of Sex" [30] in which I discussed various hypotheses about sex, and proposed one of my own. In the same work I discuss holism at some length. Holism seems to me perfectly legitimate if it means recognizing what goes on at higher levels. However, there are some problems when it means attributing properties to wholes which they do not really have. I note that you are aware of my work on species (and other things) as ontological individuals. I discussed many implications of the "individuality thesis" in my 1997 book "Metaphysics and the Origin of Species" [55]. When we say that species are individuals we mean that they are individuals in a strong, ontological sense, as are organisms, but it is not just an analogy. I frankly do not like the idea of calling species superorganisms because I would not call molecules superatoms. So I refer to species and some other entities as "supraorganismal wholes."

***Authors' response: ****In the revised manuscript I made changing in accord with your (and of others) comments (including more adequate citation of yours and of others works). For me, the main cognitive meaning of holism is in understanding that an organized whole always has some properties that cannot be reduced to or induced from the properties of the parts. Moreover, the essence of a whole is always among these irreducible properties*.

*Reviewer 2*

Sex is important in speciation theory because it bestows cohesiveness on populations. I much prefer to say cohesiveness rather than integration. There is a problem with the term "population" that you should watch out for in this context. When we are discussing species as reproductively isolated populations the populations are sexual by definition - wholes held together by sex. Talk about asexual populations suggests that they are mere samples or arbitrary sex. There are serious problems with treating sex as if it were something that occurs only in eukaryotes, though eukaryote sex is different in some ways. Prokaryotes have more sex than was previously realized and they do form species. Although as you say the generality of eukaryotes are sexual, your audience may need to be reminded that there are important exceptions.

***Authors' response: ****There are numerous variants of sexual reproduction among eukaryotes, which I was not going to discuss. I deliberately limited myself with obligatory sexual reproduction where its meaning looks most clear. Bacteria have genetic exchanges but now and then. They have sex but not sexual reproduction*.

*Reviewer 2*

The idea that sex and selection are conservative is a familiar one and a good point. Schmalhausen's idea of stabilizing selection comes to mind. You say that Darwinian selection occurs only at the organismal level. Actually Darwin believed in selection at the level of the family.

***Authors' response: ****You are right. I just cited Wikipedia. Darwin was an open-minded man. Still he construed individual organisms as most important units of selection*.

*Reviewer 2*

Much of what you say about Dawkins talk is agreeable to me. I and others have said as much before. The trouble with talk about group versus individual selection is that all sorts of things are both individuals and groups. Every molecule is an individual molecule and also a group of atoms. Things are much more clear when one says organism whenever that is what one means by an individual.

***Authors' response: ****Throughout this paper, I say organism when I mean "autonomous individual organism" be it bacterium, monocell eukaryote or multicell eukaryote. I am afraid not to be understood by the community if I name "organism" a population or species. So let species be named species though it is certainly individual too. I think the trouble with group selection is the trouble only for those who do not like this idea because even gene is a group of base pairs. But of course, when speaking about group selection, one should imply the group as organized whole. In case of sexual organisms the unit of selection is not group but "generation of a group", the group being "units of evolution". Group evolves while generation changes*.

*Reviewer 2*

On page 32 you say that a part cannot control a whole. What about a general in command of an army?

***Authors' response: ****In the revised manuscript, I discuss this problem in more detail*.

*Reviewer 2*

It seems that my comments have been of some use to you. I will be interested in seeing the published version.

*Reviewer 3*

Emanuel Tannenboum, Department of Chemistry, Ben-Gurion University of the Negev, Israel

The paper presents a number of interesting speculations regarding the purpose of sex and the role of speciation in biology. Given that my work is mainly in mathematical biology, I am generally used to reviewing papers that present a specific mathematical model. I am not used to reviewing "big picture" speculative works such as yours, though I have published a few such works myself, and I believe that they have an important role to play in the scientific literature.

Thus, given my relative lack of expertise in reviewing such papers, I am willing to defer to the opinions of the other two reviewers, who believe that the manuscript merits publication.

My main suggestion for improving the manuscript is to remove or re-word certain parts of the paper that deal with philosophical interpretations of the second law of thermodynamics, or that misapply this law (which is very common). Specifically, you discuss death as the inevitable victory of the second law of thermodynamics over living systems, and that living systems apparently go against the second law. This is not true. The entropy of a given system can decrease, as long as it is coupled to a corresponding entropy increase somewhere else. The second law states that the entropy of an isolated system must always increase. However, living systems are not isolated. They take in external resources from the environment, and these resources produce energy via chemical reactions that sustains the chemical reactions necessary for maintaining the living system. In this context, the energy flow into the living system is what allows living systems to avoid increasing their entropy, and this is completely compatible with the second law.

The origin of death does have to do with decay of a system, but the mechanisms leading to aging and death are more complicated than a simple invocation of the second law. One theory is that aging and death is caused by the steady decay of the stem cell population in an adult organism, due to various damaging agents whose effect accumulates over time. Eventually, the organism does not have a sufficient number of stem cells to properly renew damaged or dead tissues, and the eventual result is organ failure and death.

***Authors' response: ****My way of using thermodynamic terminology and interpretation of the second law is clarified in my response to the reviewer 1. In the revised version, I tried to further improve the wording to avert the danger of misunderstanding. I stress that I never stated that "living systems go against the second law". I am sure they do not. They are systems that are far from thermodynamical equilibrium, i.e. they are low-entropy systems. And I completely agree that"the energy flow into the living system is allows living systems to avoid increasing their entropy, and this is completely compatible with the second law". But this general true is only physics. Energy in itself is ****chaotizing factor***. *Biological sciences (genetics, morphology, biochemistry, physiology, embryology, ethology et cetera) are to explain ****how****the living systems can use energy and substance to prevent entropy growth. And evolution theory is to explain how such "antientropic" systems can appear and persist in nature. In the same way, death as thermodynamic equilibrium is true statement but it is only physics. It is for biology to find out and explain numerous concrete causes of death and extinction. This paper is not about thermodynamics. My addressing to entropy is partially explained by necessity to express my opinion that the "struggle for existence" is not so much competition between the individuals of the same species as improvement of their own organization, their ability to resist entropy*.
